# Comparative Transcriptomes of the Body Wall of Wild and Farmed Sea Cucumber *Isostichopus badionotus*

**DOI:** 10.3390/ijms22083882

**Published:** 2021-04-09

**Authors:** Roberto Martín-Hernández, Rossanna Rodríguez-Canul, Nuvia Kantún-Moreno, Miguel A. Olvera-Novoa, Oscar Medina-Contreras, Cristobal Garikoitz-Legarda, Juan Carlos Triviño, Jesús Alejandro Zamora-Briseño, Víctor May-Solis, Alicia Poot-Salazar, Juan Antonio Pérez-Vega, Judit Gil-Zamorano, George Grant, Alberto Dávalos, Leticia Olivera-Castillo

**Affiliations:** 1 Bioinformatics and Biostatistics Unit, IMDEA Food Institute, CEI UAM+CSIC, Carretera de Cantoblanco 8, 28049 Madrid, Spain; roberto.martin@imdea.org; 2 Laboratorio de Inmunología y Biología Molecular, Centro de Investigación y de Estudios Avanzados del IPN-Unidad Mérida, Antigua Carretera a Progreso Km. 6, Mérida 97310, Yucatán, Mexico; rossana.rodriguez@cinvestav.mx (R.R.-C.); nuviakm@gmail.com (N.K.-M.); zambri33@hotmail.com (J.A.Z.-B.); juan.vega@cinvestav.mx (J.A.P.-V.); 3 Laboratorio de Nutrición Acuícola, Centro de Investigación y de Estudios Avanzados del IPN-Unidad Mérida, Antigua Carretera a Progreso Km. 6, Mérida 97310, Yucatán, Mexico; miguel.olvera@cinvestav.mx (M.A.O.-N.); victormay_29@hotmail.com (V.M.-S.); 4 Unidad de Investigación Epidemiológica en Endocrinología y Nutrición, Hospital Infantil de México “Federico Gómez”, Mexico City 06720, Mexico; omedina@himfg.edu.mx; 5 Bioinformatics Department, Sistemas Genómicos S.L., Ronda de Guglielmo Marconi 6, 46980 Paterna, Spain; garikoitz.legarda@sistemasgenomicos.com (C.G.-L.); jc.trivino@sistemasgenomicos.com (J.C.T.); 6 Centro Regional de Investigaciones Acuícola y Pesqueras en Yucalpetén, Instituto Nacional de Pesca y Acuacultura, Boulevard del Pescador S/N, Puerto de Abrigo, Progreso 97320, Yucatán, Mexico; alipootsalazar@gmail.com; 7 Laboratory of Epigenetics of Lipid Metabolism, IMDEA Food Institute, CEI UAM+CSIC, Carretera de Cantoblanco 8, 28049 Madrid, Spain; judit.gil@imdea.org; 8 School of Medicine, Medical Sciences and Nutrition, University of Aberdeen, Aberdeen AB25 2ZD, UK

**Keywords:** gene expression, RNA-sequencing, holothurids, transcriptome, sea cucumber, aquaculture

## Abstract

Overfishing of sea cucumber *Isostichopus badionotus* from Yucatan has led to a major population decline. They are being captured as an alternative to traditional species despite a paucity of information about their health-promoting properties. The transcriptome of the body wall of wild and farmed *I. badionotus* has now been studied for the first time by an RNA-Seq approach. The functional profile of wild *I. badionotus* was comparable with data in the literature for other regularly captured species. In contrast, the metabolism of first generation farmed *I. badionotus* was impaired. This had multiple possible causes including a sub-optimal growth environment and impaired nutrient utilization. Several key metabolic pathways that are important in effective handling and accretion of nutrients and energy, or clearance of harmful cellular metabolites, were disrupted or dysregulated. For instance, collagen mRNAs were greatly reduced and deposition of collagen proteins impaired. Wild *I. badionotus* is, therefore, a suitable alternative to other widely used species but, at present, the potential of farmed *I. badionotus* is unclear. The environmental or nutritional factors responsible for their impaired function in culture remain unknown, but the present data gives useful pointers to the underlying problems associated with their aquaculture.

## 1. Introduction

Sea cucumbers (Phylum Echinodermata, Class Holothuroidea) are a diverse group of benthic marine invertebrates that inhabit intertidal to abyssal zones in temperate and tropical oceans worldwide [1]. There is a high demand for them in Asian markets, where sea cucumber are widely used as a luxury seafood and in traditional medicines. They are also a rich source of potential health-promoting factors such as fucosylated chondroitin sulphates, collagens, peptides, and sphingolipids, among others [2,3]. Overall, this has led to over-fishing of wild stocks and depletion of some species [4], including *Isostichopus badionotus* from the Caribbean Sea and Gulf of Mexico. In fact, capture of *I. badionotus* from off the coast of the Yucatan State has been so intense in recent years that federal regulatory authorities have implemented protective measures to prevent the regional extinction of the species [5,6]. This was vital not only to preserve stocks but also to protect the local marine environment since sea cucumbers recycle nutrients and break down detritus and other organic matter on the sea bottom and have a critical role in maintaining the marine ecosystems [4,7].

Farming of *I. badionotus* could in part alleviate this problem by offering a readily available commercial source of mature sea cucumbers or alternatively by providing seed animals for restocking and regeneration of wild populations. Cultivation studies are ongoing [8,9,10]. In these, animals are kept in indoor fiberglass tanks in a closed recirculating system with a temperature maintained at a range between 24–26 °C and a 13:11 (light:dark) photoperiod [8]. The main feed offered is macroalgae-based, with most diets including a mixture of *Ulva* sp., *Sargassum* sp. *and Macrocystis* sp., with fatty acid-rich supplements [8]. Under these conditions most *I. badionotus* survive, go through larval development, and slowly grow and mature as juveniles and adults [8,9,10].

Much of the therapeutic and commercial potential of sea cucumber lies in the range of health-modulating factors that are expressed by it [2,3]. However, the profile, yield, and reactivity of constituent bioactive factors are dependent on the exact metabolic state of the sea cucumber and individual compounds may be reduced, lost, or produced in inactive forms if host metabolism is disturbed. While *I. badionotus* has been shown to grow in culture, little is known about its metabolic state during cultivation and hence its capacity to produce functional health-promoting components.

Omics approaches, such as high-throughput RNA sequencing and subsequent bioinformatics analyses, have provided useful insights into the biology of sea cucumber species. For example, the de novo transcriptomic assembly of *A. japonicus* has identified key genes associated with important traits such as disease response, color variation, growth, and development [11,12,13,14]. 

In the present study, the transcriptome of body wall tissue from *I. badionotus* from Yucatan has been described for the first time. In addition, a comparison between the transcriptomes of wild (captured) and farmed age-equivalent *I. badionotus* has been done to assess the similarity/dissimilarity in their metabolic profiles.

## 2. Results

### 2.1. Samples

Adult *Isostichopus badionotus* (Holothuria) were collected off the coast of the state of Yucatan, Mexico. The organisms were placed in individual plastic bags containing sea water from the collection site and kept at 22 to 24 °C, the average water temperature at the collection site during transport to the laboratory. The individual lengths and body weights were measured (Supplementary Table S1) and four representative animals (14–25 cm, 160–420 g), whose ages were estimated at approximately two years by the procedures of Poot-Salazar et al., 2014 [15], were selected for transcriptome analysis.

*I. badionotus* brood-stock had been collected previously from the same location and transported to the Sea Cucumber Aquaculture Laboratory, Telchac Puerto Marine Station, CINVESTAV. The animals were kept in indoor fiberglass tanks at 24–26 °C under a photoperiod of 13:11 (light:dark), to simulate summer conditions and fed algae-based meals in accordance with recommended procedures [8].

Brood-stock maintained under these culture conditions spawned spontaneously during the normal reproductive season of July to November without induction [9]. When spawning occurred, gametes were collected for artificial fertilization and incubated in 250 L fiberglass tanks. Larval feeding was done with live microalgae produced in situ, and commercial microalgae concentrates [8,9]. 

Early juveniles were transferred to fresh fiberglass tanks for subsequent growth. They were fed a mix of algae supplemented with periodic additions of live and concentrated diatoms, following published protocols [10]. Four representative first generation individuals of approximately two years of age were taken for transcriptome analysis (5–7 cm, 17–43 g) (Supplementary Table S1).

### 2.2. Transcriptome Sequencing

Eight cDNA libraries were generated from the *I. badionotus* body wall mRNA for comparative transcriptomic analysis between the two sea cucumber groups: wild adult (~two-year-old) specimens (4 cDNA libraries) and two-year-old farmed animals born and reared in captivity (*n* = 4). 

Total RNA was extracted from individual body wall samples using TRIzol Reagent. Preparations with an RNA integrity number (RIN) >7.0 were taken forward for cDNA library preparation. mRNA isolation, cDNA synthesis, and library preparation were done using the TruSeq Stranded mRNA Sample Preparation Kit. The quality and size distribution of the cDNA libraries was validated using a TapeStation Genomic DNA system. 

The libraries were processed with Illumina deep-sequencing. After paired-end sequencing, a total of 320,540,514 raw reads with a 76 bp average length were acquired, with four libraries corresponding to wild adult specimens: 74,199,152 (Wild1), 83,116,920 (Wild2), 77,041,560 (Wild3) and 86,182,882 (Wild4). These were used for de novo transcriptome assembly. Raw data quality is described in Supplementary Table S2. The raw reads were deposited in the Sequence Read Archive (SRA) in the GenBank database, linked to BioProject PRJNA639785 (https://www.ncbi.nlm.nih.gov/bioproject/PRJNA639785 accessed on 16 June 2020), which also includes the sequences resulting from transcriptome assembly. A total of 293,055,064 raw reads with a 150 bp average length were obtained from farmed individuals: 39,511,252. The raw reads from the farmed individuals were used to evaluate differential gene expression and are available in the GEO database with ID GSE157183 (https://www.ncbi.nlm.nih.gov/geo/query/acc.cgi?acc=GSE157183 accessed on 1 September 2020). 

### 2.3. De Novo Transcriptome Assembly

**Figure 1:**
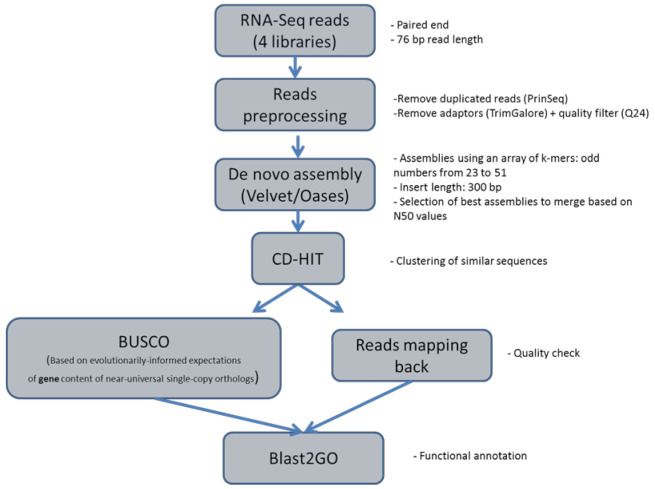
Bioinformatics pipeline used for transcriptome de novo assembly.

Processed paired-end reads obtained from the wild sea cucumber specimens were used for de novo assembly. As low k-mer values are prone to introduce false contigs, and it is recommended to use a maximum k-mer size corresponding to 2/3 of the read length to allow enough overlapping, a range of k-mers between 23 and 51 was tested in order to capture short and longer transcripts with sufficient coverage (Figure 1). Following selection of the best assemblies based on N50 values (Supplementary Table S3), and merging and filtering contigs shorter than 100 bp, the merged assembly produced with Velvet included 148,431 contigs. Contig size ranged from 101 to 6957 bp, with a 384.9 bp average length (Supplementary Figure S1A). The relationship between contig size and contig coverage exhibited a positive trend, with a 5.52 mean coverage value (Supplementary Figure S1B).

Oases was used for the scaffolding step. Therefore, after filtering redundant sequences exploiting pairing information of reads to further connect contigs, and producing transcript isoforms, the final assembly was reduced to 132,257 consensus sequences, representing a total of 81,784 unique loci or unigenes (Supplementary Table S4). Average unigene length was 679 bp, with a maximum of 10,560 bp and a N50 value of 1219.

Quantitative measurement of transcriptome assembly process completeness was done with the BUSCO software, which is based on sets of Benchmarking Universal Single-Copy Orthologs from OrthoDB [16]. The utilized lineage-specific library corresponds to Eukaryota. We were able to completely recover a total of 278 BUSCO genes, thus achieving a 65% of completeness. In addition, this tool was used to compare our de novo transcriptome assembly with two other sea cucumber species belonging to *Apostichopus japonicus* transcriptome shotgun assemblies (Acc. IDs: GFKU00000000.1 and GHCH00000000.1) available online on Genbank, part of the NCBI Nucleotide database.

**Figure 2:**
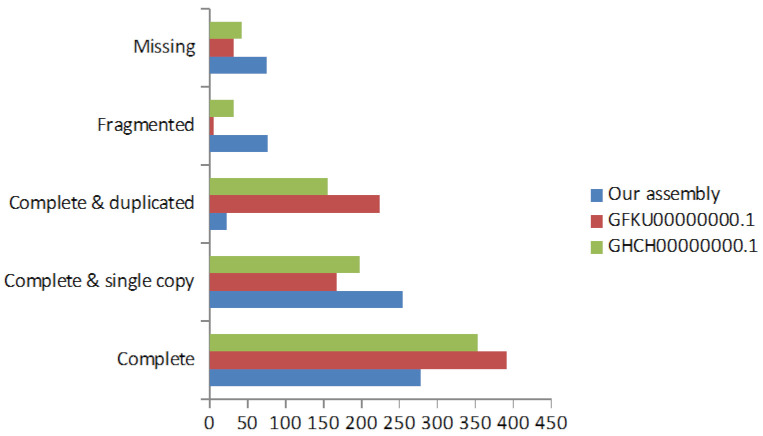
Comparison of BUSCO results among three assemblies. Quantitative assessment of transcriptome completeness for the newly assembled wild cucumber transcriptome, and comparison with related publicly available transcriptome assemblies.

Two of the GenBank assemblies slightly outperformed our assembly in terms of complete identified BUSCOs (Figure 2), but neither was able to recover the full set of 429 eukaryote orthologs. Our assembly also exhibits a significant reduction in the number of duplicated BUSCOs, probably due to removal of duplicated reads during the de novo assembly process. Further differences may be due to the tool chosen for de novo assembly, since the two GenBank assemblies were produced with the Trinity de novo assembly tool [17].

### 2.4. Transcriptome Annotation

**Figure 3:**
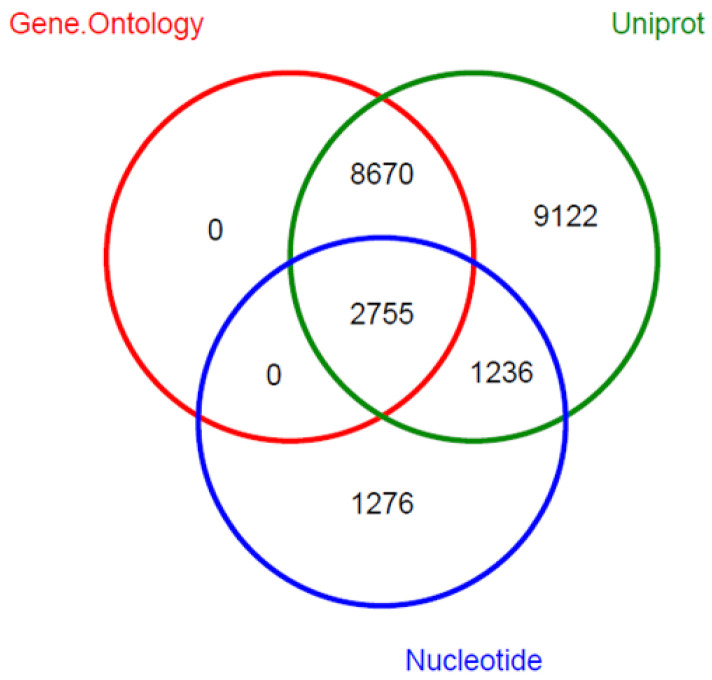
Distribution of annotated unigenes across databases. Venn diagram for common annotated unigenes in NT, UniProt and Gene Ontology databases.

BLAST analyses of the 132,257 non-redundant assembled transcripts were run against different databases. A total of 5275 assembled transcripts had hits with 3599 unique sequences from the NT database, while 21,783 had hits with 12,609 unique protein sequences from the Uniprot database. GO functional annotation resulted in annotation of 11,425 unique transcripts with 7390 highly specific GO terms and KEGG enzyme IDs (Figure 3). Comparing these results to those reported by a previous transcriptome assembly of a closely related species [14], the amount of functionally annotated genes against GO database is very similar. However, the above-mentioned study reported higher annotation rates against NT and Uniprot databases. This fact might be explained by the low level of gene duplication and higher gene missingness of the transcriptome assembly generated in this research (Figure 2), as well as by the use of a different assembler.

RNAcentral analysis produced 218 potential RNAs, of which 84 corresponded to ribosomal RNAs, 80 to long non-coding RNAs, 34 to miscellaneous RNAs, 8 to transfer RNAs and 4 to antisense RNAs. A small fraction was identified as small nucleolar RNAs (*n* = 3). One RNA was a precursor and another a bacterial transfer-messenger RNA, while three sequences remained as unclassified RNAs (data not shown)

### 2.5. Gene Ontology (GO) Classification

**Figure 4:**
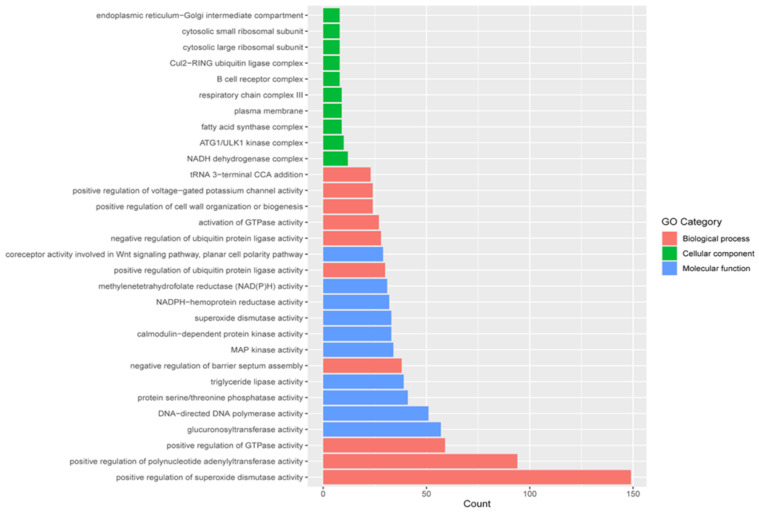
Gene Ontology (GO) classification of wild sea cucumber body wall assembled transcripts.

Assigned GO terms for the wild sea cucumber assembled transcriptome were distributed among highly specific biological processes (3071), molecular functions (2077), and cellular components (736) (Supplementary Table S5). Among the biological processes, the top three most frequent terms were GO:1901671 (“positive regulation of superoxide dismutase activity”) (149), GO:1904247 (“positive regulation of polynucleotide adenylyltransferase activity”) (94) and GO:0043547 (“positive regulation of GTPase activity”) (59). For molecular functions, GO:0015020 (“glucuronosyltransferase activity”) (57), GO:0003887 (“DNA-directed DNA polymerase activity”) (51) and GO:0004722 (“protein serine/threonine phosphatase activity”) (41) were the most frequent terms. For cellular components, GO:0030964 (“NADH dehydrogenase complex”) (12), GO:1990316 (“ATG1/ULK1 kinase complex”) (10) and GO:0045275 (“respiratory chain complex III”) (9) were the most frequent (Figure 4).

### 2.6. Differentially Expressed Genes between Wild and Farmed Sea Cucumbers

Sequenced reads from each individual were mapped to the reference transcriptome to quantify gene expression of body wall samples in both groups (Table 1). In addition, reads obtained from wild specimens were mapped against the two previously tested transcriptome assemblies (GFKU00000000.1 and GHCH00000000.1), and against a publicly available *Apostichopus japonicus* genome assembly (ASM275485v1), thus obtaining spurious overall alignment rates (Supplemental Table S6). Results from this comparative analysis highlight a significant genomic difference between sea cucumbers from the *Apostichopus* and *Isostichopus* genus. Furthermore, a sample (W2) showing the higher alignment rate on ASM275485v1 genome assembly was selected to evaluate the most highly expressed genes which were detected (Supplemental Table S7).

In transcript counting per analyzed sample, only reads showing a unique match to the transcriptome reference were counted as valid alignments (MAPQ = 60). For differential expression assessment, only transcripts detected in both groups were considered for differential expression.

**Figure 5:**
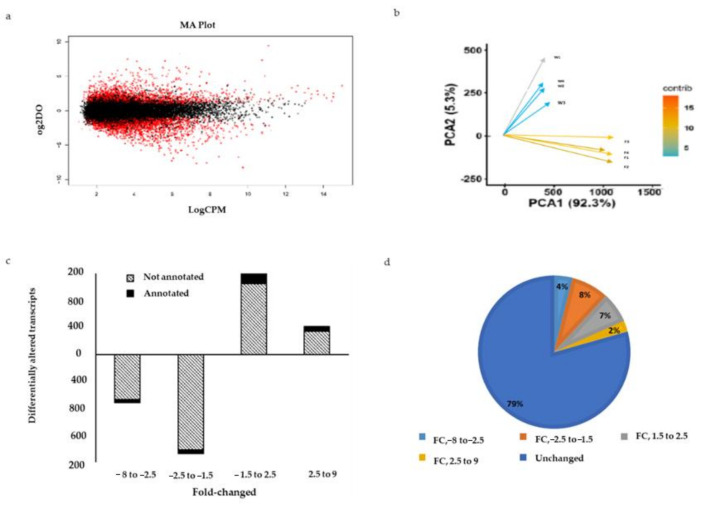
Transcriptomic comparisons between wild and farmed sea cucumbers. (**a**) MA plots of differentially expressed genes (DEGs) between wild and farmed sea cucumbers. Red dots show transcripts with a false discovery rate (FDR) < 0.05. (**b**) PCA and variable contribution to the transcriptomic data. (**c**) Differentially altered transcripts. (**d**) Percentage of altered transcripts.

Of the 132,257 newly assembled consensus transcript sequences from wild *I. badionotus*, consistent expression of 18,449 transcripts was detected across all the analyzed samples from both groups. From these, a total of 3760 transcripts (20.4%) were found to be differentially expressed between the wild and farmed groups (Figure 5a); the False Discovery Rate threshold was significant (FDR < 0.05) and a log2 fold change threshold was used. Of these transcripts, only 303 were known sequences showing a hit in the NT database (Supplementary Table S8). Gene names for each annotated transcript were retrieved when available. Principal components analysis (PCA) clearly separated the farmed (reared in culture or maintained in culture after capture) from the wild animals (Figure 5b). The contribution of each variable and the samples to the PCA are shown in Supplementary Figure S1.

The identified differentially expressed genes (DEGs) were mapped against the KEGG KO (KEGG Orthology) database, which contains molecular functions represented in terms of functional orthologs. The identified genes were mapped to 83 pathways (table KEGG_ko mapping, Supplementary Table S9), which are implicated in organism development and growth. 

Several annotated genes involved in host responses to metabolic stress, including COXI, EIF2AK3, ATPA, MAPK14-1, HSP40, hsc70, and TRPM2, were upregulated in farmed *I. badionotus* (Table 2). The EIF2AK3 gene mapped to the Mitophagy pathway (ko04137). 

Of the annotated downregulated transcripts, Techylectin 5A, CREB1 and collagen mRNAs, were of particular relevance (Table 2). Sea cucumber lectins are involved in host immune responses to infection, inflammation, cell-cell or cell-extracellular matrix interactions and self- recognition [18,19]. If lectin expression was low in the farmed animals, they were likely to be prone to immunological compromise and disease. CREB1 is a gluconeogenesis regulatory factor and linked to the Longevity regulating pathway. Collagen is a major component of the body wall of sea cucumber and any reduction in its synthesis, as suggested by the under-expression of collagen mRNAs in farmed *I badionotus*, is likely to greatly impair their health and well-being.

Preliminary collagen extraction studies done on body wall from wild and farmed *I. badionotus* do indeed indicate that the constituent collagens differ significantly (Olivera-Castillo, unpublished data). Collagen from farmed animals was more fibrous and difficult to extract by standard procedures [20] than that from wild counterparts and was of reduced proportion of body wall mass. 

### 2.7. Functional Enrichment between Wild and Farmed Sea Cucumbers

**Figure 6:**
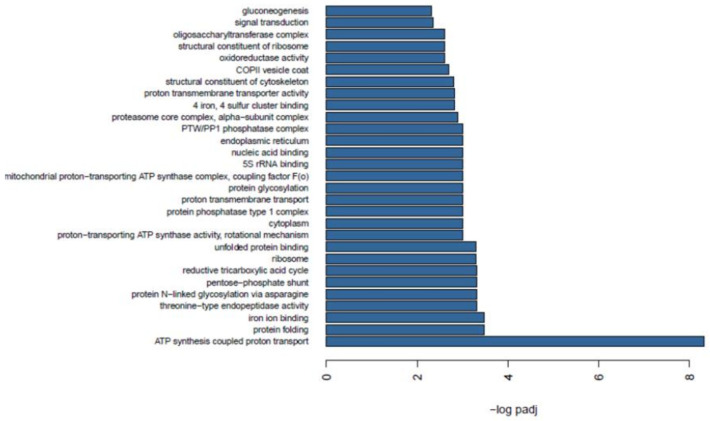
Gene Ontology (GO) analysis of sea cucumber differentially expressed genes modulated in farmed animals.

Enrichment analysis allows identification of overrepresented functional categories among differentially expressed transcripts. Some of the most important biological processes and molecular functions induced by captivity (Figure 6) were ATP synthesis (GO:0015986), proton-transporting ATP (GO:0046933), proton transport (GO:0015992) and the oxidoreductase process (GO:0016705). These functions are associated with energy synthesis for organism growth and proliferation. Other interesting significant functions were gluconeogenesis (GO:0006094), cytoskeleton (GO:0005200), and the vesicle process (GO:0030127). Other biological processes related to protein synthesis included protein folding (GO:0006457), protein glycosylation (GO:0006486), protein N-linked glycosylation via asparagine (GO:0018279), and unfolded protein binding (GO:0051082).

### 2.8. RT-qPCR Validation of Differentially Expressed Genes Induced by Captivity

**Figure 7:**
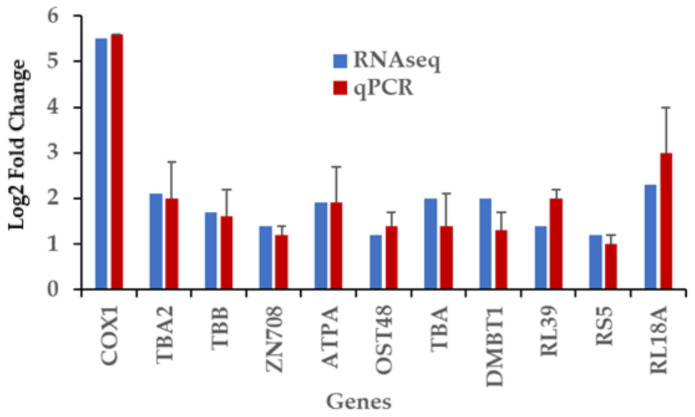
Reverse-transcription real-time PCR validation of eleven genes differentially modulated by captivity. COX1, cytochrome c oxidase subunit 1; TBA2, tubulin α chain 2; TBB, Tubulin β chain; ZN709, zinc finger protein 709; ATPA, ATP synthase subunit α; OST48, dolichyl-diphosphooligosaccharide-protein glycosyltransferase non-catalytic subunit; TBA, tubulin alpha chain; DMBT1, deleted in malignant brain tumors 1; RL39, ribosomal protein L39; RS5, ribosomal protein S5; RL18A, ribosomal protein L18a. Data is expressed as relative expression compared to that of wild sea cucumbers (*n* = 5).

We validated the DEG results produced in the wild vs. farmed (reared in culture) sea cucumber comparisons by sampling five sea cucumbers from the coast of the Yucatan state. We randomly selected eleven genes (oligo sequences in Supplementary Table S10) from the 303 transcripts (NT database hits) for validation analysis by RT-qPCR. In most cases, the RT-qPCR results exhibit behavior similar to the RNA-seq analysis (Figure 7), suggesting that our de novo assembly of the *I. badionotus* transcriptome is reliable.

## 3. Discussion

Sea cucumber (*Isostichopus badionotus*) is vital for maintaining the marine microenvironment and water quality due to its ability to scavenge calcareous algae, foraminifera, molluscs, and clear sedimentary detritus from the seabed [8,20]. However, a rapid decline in *I. badionotus* stocks from Yucatan’s coast due to overfishing has seriously compromised this ecological protection [4,5]. This exploitation of *I. badionotus* is due to its use as a substitute luxury seafood and as a potential source of health-promoting factors to replace traditional sea cucumber species whose wild stocks are already depleted [2,3,5,6]. Despite the high usage of *I. badionotus*, little is known about its general metabolism or indeed, its comparability to other sea cucumber species. To address this in part, the functional transcriptome of the body wall of wild-type *I. badionotus* from Yucatan has herein been described for the first time and compared with data available in the literature for other sea cucumber species. 

The transcriptome of wild-type *I. badionotus* yielded 132,257 consensus sequences, representing a total of 81,784 unique loci or unigenes. From these, 5275 assembled transcripts had hits with 3599 unique sequences from the NT database, while 21,783 had hits with 12,609 unique protein sequences from the Uniprot database. GO functional annotation identified 11,425 unique transcripts with 7390 highly specific GO terms and KEGG enzyme IDs. Amongst the biological processes, molecular functions, and cellular components identified those linked to cellular respiratory chain complex activity, positive regulation of cell growth and protection from generated reactive oxygen species were prominent. The transcriptome of wild *I. badionotus* had a high degree of similarity with that for *Apostichopus japonicus* [12,13,14,21,22,23,24], and *Stichopus horrens* [25] and the proteome reported for *Stichopus japonicus* [26]. The immune molecule progenitors identified in wild *I. badionotus* also closely matched those reported for other sea cucumber species [27,28]. 

Overall, the functional metabolic profile of wild *I. badionotus* from Yucatan is comparable with that of *A. japonicus* and other captured species. Recent studies have also shown that *I. badionotus* contains bioactive factors, such as glycosaminoglycans, peptides, saponins, and lectins, which have health-modulating properties comparable to those products from other sea cucumber species [2,3,29,30]. Therefore, *I. badionotus* from Yucatan is a proper alternative to traditionally used sea cucumber species. However, its ongoing capture from the wild is non- sustainable. To supply the commercial demands for this species a viable alternative to fishing, such as aquaculture, is needed. 

Studies on farming of *I. badionotus* are ongoing [9,10]. However, as with many sea cucumber species [8,20,31,32], rearing *I. badionotus* in culture has proved problematic with development and growth being variable and slow. Here we now show that the active transcriptome of farmed *I. badionotus* was much altered compared to its wild counterpart. Of 18,449 transcripts common to both sets of animals, twenty-one percent were differentially expressed in farmed *I. badionotus* (4% downregulated by 2.5 to 8.0-fold and 8% by 1.5 to 2.5-fold; 7% upregulated by 1.5 to 2.5-fold and 2% by 2.5 to 9.0-fold). 

The farmed *I. badionotus* studied here were first generation offspring derived from *I. badionotus* brood-stock collected from the same location off the coast of the state of Yucatan as the wild animals that were tested. The differential changes in their transcriptome were, therefore, due mainly to adaptational changes to the laboratory environment in which they were raised rather than longer-term effects of domestication or selection. Indeed, the findings of a recent study [33] indicate that these growth-limiting changes may be reversed if farmed juveniles are transferred to sea shelters close to the area of parental capture. In this case, growth of the juveniles was greatly increased compared to that laboratory environment [33]. This would be consistent with a previous report of little molecular variance between sea-based wild and cultured *A. japonicus* populations [34].

The present study’s data demonstrates that multiple aspects of normal host metabolism were disrupted in farmed *I. badionotus*. In the wild, the local environment, including season, pressure, temperature, light, salinity, and nutrient and micronutrient availability, greatly influence sea cucumber physiology and development [4,12,13,14,35,36,37]. While farmed *I. badionotus* were reared under conditions that approximated those off the Yucatan coast and received appropriate nutrients, our present findings indicate that some of these environmental or nutritional parameters were sub-optimal. Indeed, some of the differential changes observed could be associated with low pressure, intense lighting, inappropriate habitat, stress, microbiota, as well as nutrient or micronutrient deficiencies in the laboratory setting [12,38,39,40,41].

Major deregulated processes included the Mitophagy pathway, the Longevity regulating pathway and ATP synthesis coupled proton transport. Of the proportion of the transcripts that were annotated, the most significant changes were in collagen mRNAs (−4–5 to −5.8-fold) and cytochrome c oxidase subunit I (COI) gene (+5.5 to +9.0-fold).

The EIF2AK3 gene of the Mitophagy pathway was overexpressed in farmed *I. badionotus*. Mitophagy is the process by which damaged or stressed mitochondria are degraded and cleared by autophagy. EIF2AK3 is linked with the inactivation of the eukaryotic translation-initiation factor’s alpha subunit, reduced translational initiation, and repression of global protein synthesis [42]. In contrast to EIF2AK3, the gluconeogenesis regulatory gene CREB1 [43] of the Longevity regulating pathway was downregulated in the farmed animals.

Mitochondrial function and viability may be significantly impaired in farmed *I. badionotus*. In addition to EIF2AK3, cytochrome c oxidase subunit 1 (COX1) and ATP synthase subunit alpha (ATPA), which are involved in the mitochondrial inner membrane electron transport chain were also upregulated. This change may be associated with the accumulation of reactive oxygen species, cytoplasmic acidification, caspase activation, and cell death [44]. 

Collagen mRNAs were considerably downregulated in farmed *I. badionotus*. Since collagen is the major component (~70%) of the body wall of sea cucumber [45], this finding indicates that collagen production was significantly impaired or suppressed in these cultured animals. Preliminary extraction studies tend to confirm this in that the collagen of farmed *I. badionotus* was more fibrous, less readily extractable and a lower proportion body wall mass than that from wild counterparts. 

The diet offered to farmed *I. badionotus* was formulated per present knowledge of their requirements [8,9,10]. However, it cannot be excluded that important, yet unidentified nutrients or micronutrients were missing or deficient in the food. Alternatively, the digestion, absorption, and systemic processing/utilization of nutrients and micronutrients, including important organic and inorganic compounds, may be impaired due to modifications to digestive enzyme activity in the intestine and the constituent bacterial and fungal populations resulting from the culturing conditions [46,47,48,49,50,51]. 

Alternatively, the capacity of farmed *I. badionotus* to deal with and clear toxins and other deleterious cellular factors and metabolites may be compromised. The increased activity of ATP-dependent glucuronosyltransferases, which are responsible for the formation of glucuronides with a large variety of cytotoxic and genotoxic compounds and aid in their clearance [52], and increased expression of EIF2AK3 gene, which encodes a protein with critical roles in detection and initiation of cellular responses to endoplasmic reticulum stress [26], may be indicators of such disruption to systemic metabolism. The farmed animal may thus need to restrict metabolic activity to survive and remain healthy [31,53]. 

The transcriptome data presented here show the functional metabolism in wild *I. badionotus* from Yucatan was like that of *A. japonicus* and other widely captured sea cucumber species. However, when *I. badionotus* was raised and maintained in culture its metabolism was significantly disrupted and production of at least one major body component impaired. The exact factors responsible for these adverse changes in farmed *I. badionotus* and their modes of action need further detailed study particularly considering the urgent need to find a sustainable alternative to capture from the wild to supply the commercial market. 

## 4. Materials and Methods 

### 4.1. Collection of Wild Sea Cucumbers

Adult *Isostichopus badionotus* (Holothuria) were collected by coastal scuba diving off the state of Yucatan, México during June–July 2019. Collection was authorized via a permit (DGOPA/1009/210809/08761) issued by the National Aquaculture and Fishing Commission (Comisión Nacional de Acuacultura y Pesca).

Immediately after removal from the sea floor, the organisms were individually placed in plastic bags containing sea water from the collection site. At the surface, the bags were submerged in cold water inside coolers and kept at 22 to 24 °C, the water temperature range at the collection sites. This temperature control prevented proteolysis or autolysis. They were transported live to the laboratory. Before processing, individual length was measured with a plastic ruler in centimeter units and body weight measured with a spring scale in grams to 0.1 accuracy. This group was called “wild” sea cucumbers (14–25 cm, 160–420 g; Supplementary Table S1).

### 4.2. Cultivation of Sea Cucumbers

*Isostichopus badionotus* brood-stock had been collected from the same location and transported to the Sea Cucumber Aquaculture Laboratory, Telchac Puerto Marine Station, Center for Research and Advanced Studies (Centro de Investigación y de Estudios Avanzados—CINVESTAV). In the laboratory, the animals were kept in indoor fiberglass tanks forming part of a closed recirculating system including mechanical and biological filters, skimmer, and UV water-sterilization. Water temperature was maintained within a 24–26 °C range using chillers and air-conditioning. Laboratory photoperiod was a constant 13:11 (light:dark) to simulate summer conditions. The organisms were fed daily using a formulated mixture of algae meals (1:1:1; *Sargassum* sp.:*Ulva* sp.:*Macrocystis piryfera*) [8] mixed with washed and chlorinated beach sand. Before feeding, feces were removed by siphoning.

Brood-stock maintained under these culture conditions spawned spontaneously during the normal reproductive season of July to November without induction [9]. When spawning occurred, gametes were collected for artificial fertilization and incubated in 250 L fiberglass tanks. Larval feeding was done following Zacarías-Soto et al. [9], using live microalgae produced in situ and commercial microalgae concentrates (Instant Algae^®^, Reed Mariculture, Campbell, CA, USA). 

Early juveniles were transferred to fresh fiberglass tanks (see above) for subsequent growth. They were fed a mix of algae enriched with Algamac^®^ (Aquafauna Bio-Marine, Hawthorne, CA, USA), and supplemented with periodic additions of live and concentrated diatoms, following published protocols [8,9,10]. First generation individuals of approximately two years of age were collected for the transcriptome analysis; these were called “farmed” sea cucumbers (5–7 cm, 17–43 g) (Supplementary Table S1).

### 4.3. RNA Isolation, cDNA Library, and Illumina Sequencing

Both the wild and farmed sea cucumbers were processed by first washing with cold sterile distilled water to remove excess sea salt followed by immediate and rapid evisceration. Each body wall was cut into several pieces (approx. 1 cm^2^), and each piece immersed in 1 mL RNAlater™ stabilization solution (Ambion^©^, (Thermo Fisher Scientific), Mexico City, MX)^©^, Cat. AM7021), snap frozen in liquid N_2_ and stored at −80 °C until use. Total RNA was extracted using TRIzol Reagent^®^ (Ambion^©^, Cat. 15596-026) essentially in accordance with Puch-Hau et al., 2019 [54]. Quantification of total RNA was done with a Qubit 2.0 (Thermo Fisher Scientific, Mexico City, MX, Mexico), and its quality [RNA integrity number (RIN)] assessed using an Agilent 2100 Bioanalyzer^®^ with the RNA 6000 Nano Lab Chip Kit^®^ (Agilent Technologies^©^, Santa Clara, CA, USA). Total RNA with a RIN score >7.0 was accepted for cDNA library preparation. Isolation of mRNA, cDNA synthesis, and library preparation were done using the TruSeq Stranded mRNA Sample Preparation Kit^®^ (Illumina^©^, San Diego, CA, USA). Briefly, the poly(A)+ mRNA fraction was isolated from total RNA, using poly(T) oligo attached to magnetic beads. Following purification, the RNA was fragmented and primed with random hexamers, and reverse transcribed using SuperScript II Reverse Transcriptase (Invitrogen^©^, Thermo Fisher Scientific, Mexico City, MX) with addition of actinomycin D. Second-strand synthesis was done using polymerase I and RNaseH, with replacement of dTTP for dUTP to generate the double-stranded cDNA (ds-cDNA). The ds-cDNA fragments were 3′-end adenylated and ligated to Illumina paired-end sequencing adapters. Finally, the products were purified and enriched with PCR to create the final indexed double-stranded cDNA library. The quality and size distribution of the cDNA libraries was validated using a TapeStation Genomic DNA system^®^ (Agilent^©^). Library construction and sequencing were done at the National Institute of Genomic Medicine (Instituto Nacional de Medicina Genómica—INMEGEN, México City, MX). Paired-end sequencing was conducted on an Illumina NextSeq 500 platform to generate 2 × 76 or 2 × 150 paired-end (PE) reads.

### 4.4. Short Reads De Novo Assembly

Quality of the raw reads was checked using FASTQC tools [55] to identify any deviations or problems in the sequencing process. The resulting raw reads were pre-processed by consistently trimming Illumina sequencing adaptors to discard low quality read pairs using a Q24 threshold Trim Galore v.0.5.0 tool. Duplicated reads were removed to reduce redundancy and improve assembly process computational efficiency, using the same software.

The Velvet v.1.2.1 sequence assembler tool [56] was used for de novo transcriptome assembly with a multi-k assembly pipeline. Length of k-mers ranged from 51 to 23, and the corresponding estimated insert length for each sequenced library was used as a parameter. A total of nine assemblies, obtained with higher and lower k-mer lengths, were selected based on the higher N50 values obtained, and then merged using Oases v.0.2.08 [57] to obtain the final assembly. Finally, the CD-HIT v.4.7 tool [58] was used to remove redundant transcripts, with a 90% similarity threshold.

Quantitative measures of the de novo assembled transcriptome were generated by comparing the generated assembly to previous assemblies from closely related species using the BUSCO software v.4.0.2 [59].

### 4.5. Functional Annotation

The de novo assembled transcriptome was annotated using the Basic Local Alignment Search Tool (BLAST) [60] locally, with parameters such as max target seqs set to 1, best hit overhang set to 0.1 and best hit score edge set to 0.1. Queried sequence databases were the NCBI nucleotide (NT), Uniprot and RNACentral databases [61,62]. BLAST analyses results were filtered against nucleotide and protein databases, and only hits with an e-value score equal to or lower than 0.001 retained. For sequence annotation with GO terms the annotated sequences against the Uniprot database were introduced into the Blast2Go v.5.1 tool [63]. Default parameters were used which allow a compromise between coverage and correctness of the assigned functional GO term. The predicted proteins were functionally annotated according to three different functional categories available in the GO database: biological process, molecular function, and cellular component.

### 4.6. Functional Enrichment

A functional enrichment analysis was run using the GO functional information, based on hypergeometric distribution and using the R ver. 3.2.3 statistical software. Following a Fisher’s exact test, the false discovery rate (FDR) adjusted *p*-value was set at a 0.05 cut-off.

### 4.7. Differential Expression between Sea Cucumber Body Wall Transcriptomes, and Pathway Analysis

The newly assembled transcriptome from wild *I. badionotus* was used as reference for sequence alignment. The sequenced paired-end reads from each sample of both groups (wild and farmed) were mapped against the reference transcriptome using the Hisat2 software [64] setting rna-strandness parameter to FR. Only reads showing a unique match were counted as valid alignments. The resulting count table was analyzed using the edgeR (v 3.20.9) [65] library in the R environment. After calculating normalization factors to scale the raw library sizes, exact tests were applied to evaluate differences in the mean counts for each transcript between both groups, and setting the False Discovery rate (FDR) statistical significance level below 0.05. Transcripts exhibiting a minimum expression level value of 3 CPM in at least three samples in both groups were considered as consistently expressed and were used in the differential expression analysis. Differentially expressed genes (DEGs) were mapped to their corresponding pathways using the KO (KEGG Orthology) database, a database of molecular functions represented in terms of functional orthologs [66].

### 4.8. Gene Validation

Eleven DEGs from the comparative transcriptome analysis were used for validation. Eight wild and eight farmed animals were chosen for gene validation (see Supplementary Table S10 for the complete set of primers used herein). After weighing and measuring, the animals were washed, quickly eviscerated and the epidermis removed from the body wall. The body wall was snap frozen in liquid nitrogen and quickly macerated into powder. A ≈ 100 mg sample of this powder was placed in 1 mL Trizol^®^ (Invitrogen^©^). Total RNA was isolated with RNA Direct-zol RNA (Thermo Scientific^©^). The RNA was analyzed by 1.2% agarose gel electrophoresis, quantified using a NanoDrop 2000c^®^ (Thermo Scientific) and RNA quality analyzed with the Qubit system. Genomic DNA (gDNA) was removed using the TURBO DNA-free™ Kit (Ambion^©^, Cat. AM1907) per manufacturer instructions and confirmed by PCR analysis of the 16S rRNA gene. Synthesis of cDNA was done with the RevertAid H Minus First Strand cDNA kit^®^ (Thermo Scientific^©^). The RT-qPCR was done with a Rotor Gene Q (2-plex) real-time PCR detector (Qiagen^©^, Hilden, Germany) using Quantinova SYBR Green PCR master^®^ (Qiagen^©^). Four RT-qPCR reactions were run, including non-template reactions as negative controls. Thermo-cycling conditions were 5 min at 95 °C, followed by 40 cycles of 15 s at 94 °C, and 45 s at an annealing temperature (Ta), depending on each primer pair. Dissociation curves were generated to verify amplified product specificity. The expression levels of selected DEGs were normalized by comparison to MTB2 internal reference genes. Values for Ct were exported, the fold-change was estimated as relative gene expression levels using the ddCt method, transformed to log2 scale, and compared with transcriptome data [67,68]. 

### 4.9. Ethics Statement

The capture of wild organisms was done with the authorization, participation, and supervision of scientific personnel of the National Fisheries Institute, Yucalpeten, Yucatan, Mexico (INAPESCA, CRIP-Yucalpeten). Sea cucumber handling and euthanasia comply with the Mexican Official Standard for the Care and Use of Laboratory Animals (NOM-062-ZOO-1999) and with the rules of the Internal Committee for the Care and Use of Laboratory Animals of CINVESTAV.

## 5. Conclusions

De novo transcriptomes for the body wall from adult *I. badionotus* from Yucatan captured in the wild or grown in culture have been generated for the first time. The functional metabolic profile of wild *I. badionotus* was comparable with that in the literature for *A. japonicus* and other regularly captured species. In conjunction with recent findings that *I. badionotus* contains bioactive factors, such as glycosaminoglycans, peptides, etc., which have health-modulating properties, the present data indicates that *I. badionotus* from the wild can be used as a substitute for traditionally captured sea cucumber species.

The data for first generation farmed *I. badionotus* revealed that their functional transcriptome was greatly altered compared to that of their wild counterparts. This disruption to host metabolism had multiple possible causes. Despite the animals being reared under conditions approximating those off the Yucatan coast and having received appropriate nutrition, the data indicates that aspects of their growth environment and availability or utilization of nutrients and micronutrients were sub-optimal. An important indicator of the combined adverse effects was that several key metabolic pathways that are important in effective handling and accretion of nutrients and energy, or clearance of harmful cellular metabolites were disrupted or dysregulated. For example, collagen mRNAs were greatly reduced, and collagen protein deposition was impaired. 

The nature of the environmental or nutritional factors responsible for the disruption of metabolism in farmed *I. badionotus* remains unknown, but the present data gives useful insights into the difficulties associated with aquaculture of this important species.

## Figures and Tables

**Table 1:** Summary statistics of sequence alignment process.

Sample *	Number of Paired Reads	Aligned Concordantly 0 Times	Aligned Concordantly Exactly 1 Time	Aligned Concordantly >1 Time	Overall Alignment Rate
W1	43,091,441	14,423,783 (33.5%)	21,501,037 (49.9%)	7,166,621 (16.6%)	83.90%
W2	38,520,780	15,694,808 (40.7%)	16,972,681 (44.1%)	5,853,290 (15.2%)	75.50%
W3	41,558,460	16,415,528 (39.5%)	18,538,978 (44.6%)	6,603,954 (15.9%)	76.60%
W4	37,099,576	15,847,351 (42.7%)	16,619,310 (44.8%)	4,632,915 (12.5%)	84.30%
F1	22,251,490	11,211,107 (50.4%)	8,994,180 (40.4%)	2,046,203 (9.2%)	63.60%
F2	22,603,025	10,767,121 (47.6%)	9,890,391 (43.7%)	1,945,513 (8.6%)	67.40%
F3	29,004,587	12,871,831 (44.4%)	12,775,028 (44.0%)	3,357,728 (11.6%)	67.40%
F4	19,755,626	9,614,938 (48.7%)	8,230,706 (41.7%)	1,909,982 (9/7%)	65.90%

* W = Wild; F = Farmed.

**Table 2:** Selection of transcripts differentially expressed in farmed *I. badionotus*.

Gene	Fold Change
Cytochrome c oxidase subunit I (COXI)	+6-fold
Eukaryotic translation initiation factor 2-alpha kinase 3 (EIF2AK3)	+2-fold
ATP synthase subunit alpha (APTA)	+2.2-fold
Mitogen-activated protein kinase 14 (MAPK14-1)	+2.9-fold
Heat Shock Protein-40 (HSP40)	+2.3-fold
Heat Shock protein cognate70	+1.7-fold
* TRPM2	+2.9-fold
Cyclic AMP-responsive element-binding protein 1 (CREB1)	−1.9-fold
Techylectin 5A	−2.9-fold
Suppressors of cytokine signaling 2 (SOCS2)	−2.6-fold
*I. badionotus* isolate FAO44 16S ribosomal RNA (mitochondrial)	−3.9-fold
Collagen mRNAs	~−5-fold

* Transient receptor potential cation channel subfamily M member 2-like.

## Data Availability

The raw reads for wild animals were deposited in the Sequence Read Archive in the GenBank database. Data from corresponding samples are available in the following links: Wild1 (https://www.ncbi.nlm.nih.gov/sra/SRX8584713), Wild2 (https://www.ncbi.nlm.nih.gov/sra/SRX8584714, accessed on 9 April 2021) Wild3 (https://www.ncbi.nlm.nih.gov/sra/SRX8584711, accessed on 9 April 2021), Wild4 (https://www.ncbi.nlm.nih.gov/sra/SRX8584712, accessed on 9 April 2021). BioProject PRJNA639785 (https://www.ncbi.nlm.nih.gov/bioproject/PRJNA639785, accessed on 17 February 2021). The raw reads from the farmed individuals were used to evaluate differential gene expression and are available in the GEO database with ID GSE157183 (https://www.ncbi.nlm.nih.gov/geo/query/acc.cgi?acc=GSE157183, accessed on 9 April 2021).

## Supplementary Materials

The following are available online at https://www.mdpi.com/article/10.3390/ijms22083882/s1. Supplementary Figure S1: Contig and PCA analysis of differentially expressed genes between wild and farmed sea cucumber Isostichopus badionotus. (A) Contig size distribution generated by Velvet. (B) Log-log plots showing the relation of contig lengths to the number of reads assembled into each. (C) Correlation among samples. (D) Multidimensional scaling plot of the analyzed RNA-Seq samples. (E) Hierarchical cluster analysis of differentially expressed genes. Supplementary Table S1: Summary of weight, length, age and life status of each Isostichopus badionotus collected for RNAseq analysis, Supplementary Table S2: Summary of data quality of sequencing data used for de-novo assembly before and after pre-processing, Supplementary Table S3: Summary of obtained assemblies using different k-mer length. Based on the best N50 values, selected assemblies for merging are highlighted, Supplementary Table S4: Summary statistics of de novo assembly for Isostichopus badionotus transcriptome and its functional annotation, Supplementary Table S5: Gene Ontology (GO) specific term frequency, Supplementary Table S6: Overall alignment rates obtained after mapping wild *I. badionotus* samples against 3 different genomic references, Supplementary Table S7: Top 10 Genes detected after mapping Wild2 sample (W2), Supplementary Table S8: Differentially expressed transcripts showing a hit with NT database. Data includes gene symbols without determined orthologs (LOC) and locus tags obtained from sequencing projects. Supplementary Table S9: KEGG_ko mapping, Supplementary Table S10: Primers used in the expression analysis by RT-qPCR.

## Abbreviations

ATPA	ATP synthase subunit alpha
BLAST	Basic Local Alignment Search Tool
COX1	cytochrome c oxidase subunit 1
CREB1	CAMP responsive element binding protein 1
DEG	Differentially expressed gene
EIF2AK3	Eukaryotic Translation Initiation Factor 2 Alpha Kinase 3
FDR	False discovery rate
GO	Gene Ontology
KO	KEGG Orthology
RT-qPCR	Reverse transcription qualitative real-time polymerase chain rection
SRA	Sequence Read Archive
