# Supplementary material for: Comparative Transcriptomes of the Body Wall of Wild and Farmed Sea Cucumber Isostichopus badionotus
Source: Int J Mol Sci. 2021 Apr 9;22(8):3882. doi: 10.3390/ijms22083882 (PMC8070510; doi:10.3390/ijms22083882)
Supplement: Supplementary file 1 [file ijms-22-03882-s001.zip › ijms-1124128.pdf]

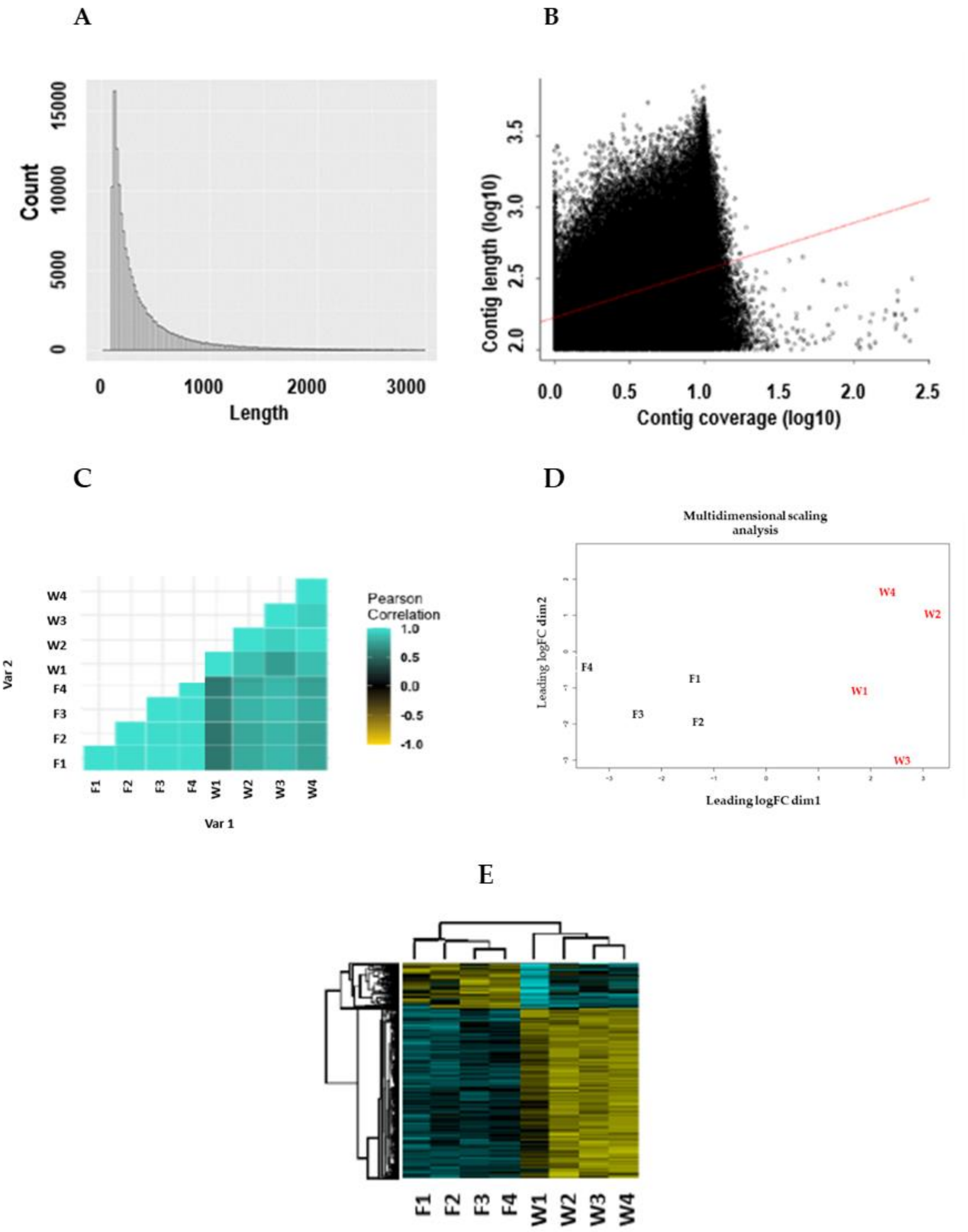

Supplementary Figure S1. Contig and PCA analysis of differentially expressed genes between wild and farmed sea cucumber *Isostichopus badionotus*.

A) Contig size distribution generated by Velvet. B) Log-log plots showing the relation of contig lengths to the number of reads assembled into each. C) Correlation among samples. D) Multidimensional scaling plot of the analyzed RNA-Seq samples. E) Hierarchical cluster analysis of differentially expressed genes.

Supplementary Table S1. Summary of weight, length, age and life status of each *Isostichopus badionotus* collected for RNAseq analysis.

**Descriptor**	**Code**	**Weight (g)**	**Length (cm)**	**Status**	**Age**
F1	P1-N	43.3	7	Farmed	2 years
F2	P4-N	24.4	7	Farmed	2 years
F3	P5-N	17.6	5	Farmed	2 years
F4	P6-N	24.6	7	Farmed	2 years
W1	P1-W	160.9	14	Wild	Adult*
W2	P8-W	420.3	25	Wild	Adult*
W3	P7-W	331.5	23.5	Wild	Adult*
W4	P2-W	164.3	14.5	Wild	Adult*

*Age assessed as approximately two year by method of Poot-Salazar et al, 2014 [20]

Supplementary Table S2. Summary of data quality of sequencing data used for *de-novo* assembly before and after pre-processing.

Sample name	Read mates	Raw reads	% Duplicated	Unique reads	% Reads with adapter	% Reads passing quality filter	Nucleotides after trimming (bp)
P1A1 (Wild1)	R1 (forward)	37,099,576	68,42	11,714,658	18,5	100	866,518,192 (98.3%)
	R2 (reverse)	237,099,576	64,25	13,261,924	19,1	100	947,949,670 (95.0%)
P2A1 (Wild4)	R1 (forward)	43,091,441	60,97	16,819,783	18,5	100	1,245,173,664 (98.5%)
	R2 (reverse)	43,091,441	57,74	18,209,179	19,5	100	1,312,172,607 (95.8%)
P7S1 (WILD3) (Wild2)	R1 (forward)	38,520,780	59,69	15,527,746	22,3	100	1,083,996,267 (93.3%)
	R2 (reverse)	38,520,780	52,05	18,470,264	17,3	100	1,202,471,304 (86.9%)
P8S16 (WILD2) (Wild3)	R1 (forward)	41,558,460	61,06	16,184,606	19,6	100	1,181,327,694 (97.5%)
	R2 (reverse)	41,558,460	52,99	19,534,631	18,1	100	1,306,416,567 (89.1%)
**Totals**		320,540,514		129,722,791			9,146,025,965

Supplementary Table S3. Summary of obtained assemblies using different k-mer length. Based on the best N50 values, selected assemblies for merging are highlighted.

Sample name	k-mer length	N50 Value
P2A1 (Wild4)	51	634
P8S16 (Wild2)	51	457
P1A1 (Wild1)	51	443
P7S1 (Wild3)	51	428
P2A1 (Wild4)	49	652
P8S16 (Wild2)	49	473
P1A1 (Wild1)	49	462
P7S1 (Wild3)	49	441
P2A1 (Wild4)	43	536
P8S16 (Wild2)	43	508
P1A1 (Wild1)	43	498
P7S1 (Wild3)	43	478
P2A1 (Wild4)	41	541
P8S16 (Wild2)	41	520
P1A1 (Wild1)	41	510
P7S1 (Wild3)	41	486
P2A1 (Wild4)	37	558
P8S16 (Wild2)	37	536
P1A1 (Wild1)	37	531
P7S1 (Wild3)	37	505
P2A1 (Wild4)	35	566
P1A1 (Wild1)	35	543
P8S16 (Wild2)	35	541
P7S1 (Wild3)	35	513
P2A1 (Wild4)	31	367
P1A1 (Wild1)	31	358
P8S16 (Wild2)	31	345
P7S1 (Wild3)	31	337
P2A1 (Wild4)	29	361
P1A1 (Wild1)	29	356
P8S16 (Wild2)	29	337
P7S1 (Wild3)	29	332
P1A1 (Wild1)	25	343
P2A1 (Wild4)	25	340
P7S1 (Wild3)	25	317
P8S16 (Wild2)	25	313
P1A1 (Wild1)	23	331
P2A1 (Wild4)	23	321
P7S1 (Wild3)	23	303
P8S16 (Wild2)	23	295

Supplementary Table S4. Summary statistics of *de novo assembly* for *Isostichopus badionotus* transcriptome and its functional annotation.

**Merged assembly**	Number of contigs	148,431
	Maximum contig length	6,957bp
	Minimum contig length	101bp
	Average contig length	384,9bp
**Final Assembly**	Number of transcripts	132,257
	Maximum transcript length	10,560bp
	Minimum transcript length	101bp
	Average transcript length	679bp
	N50 value	1,219bp
**Annotation**	Unigenes with blast hits to NT	5,275
	Unigenes with blast hits to Uniprot	21,783
	Unigenes with GO Terms	11,425

Supplementary Table S5. Gene Ontology (GO) specific term frequency. (see Excel file attached).

Supplementary Table S6. Overall alignment rates obtained after mapping wild I. badionotus samples against 3 different genomic references.

Sample	GFKU00000000.1	GHCH00000000.1	ASM275485v1
W1	0.31%	0.31%	0.29%
W2	0.98%	1.03%	0.75%
W3	0.93%	0.96%	0.56%
W4	0.53%	0.54%	0.45%

Supplementary Table S7: Top 10 Genes detected after mapping Wild2 sample (W2) reads against Apostichopus japonicus genome assembly ASM275485v1

Gene Symbol	Hits	Annotation
BSL78_17570	98	hypothetical protein BSL78_17570
BSL78_14729	79	hypothetical protein BSL78_14729
BSL78_28138	78	putative dynein heavy chain 3, axonemal-like, partial
BSL78_08597	77	hypothetical protein BSL78_08597
BSL78_21986	77	hypothetical protein BSL78_21986
BSL78_18981	76	hypothetical protein BSL78_18981
BSL78_12831	75	putative neurogenic locus Notch protein, partial
BSL78_12882	72	putative vacuolar protein sorting-associated protein 13A-like
BSL78_25012	67	putative dynein heavy chain 3, axonemal
BSL78_12572	63	putative IgGFc-binding protein

Supplementary Table S8. Differentially expressed transcripts showing a hit with NT database. (see Excel file attached)

Data includes gene symbols without determined orthologs (LOC) and locus tags obtained from sequencing projects.

Supplementary Table S9. KEGG_ko mapping. (see Excel file attached)

Supplementary Table S10. Primers used in the expression analysis by RT-qPCR.

Transcript	Sequence 5-3'	Amplicon length
**COX1_PATPE-F**	GCGTGGGCTGTTACAACTAC	133
**COX1_PATPE-R**	GCAGGAATGGTTTTGGAGCAT	
**RL18A_DANRE-F**	CAAACCCTGTCACCAGGGAA	134
**RL18A_DANRE-R**	ACGTTTGAAAATCCCGCAGC	
**RL39_DEBHA-F**	GGATTGGCCGATTCTGCTTC	163
**RL39_DEBHA-R**	CCAACACTGCCAGTAATTCCCA	
**DMBT1-MOUSE-F**	ACGACAGCCCTAAAACCCTG	153
**DMBT1-MOUSE-R**	TGTTGGAAGTGGAGTGGAGC	
**TBA_NOTVI-F**	CTTGCCAGCTCCAGTCTCAC	109
**TBA_NOTVI-R**	CTGTCTGGAGCACGGTATCC	
**OST48_XENTR-F**	CGATGTGTCAGACAAGCTGC	154
**OST48_XENTR-R**	CACACCTTAGTCGTCGCTGA	
**TBA2_PATVU-F**	AGCGGTGGATACTTGTGGTG	106
**TBA2_PATVU-R**	CGGGTCTGGTTTCTCTTCCC	
**OST48_DANRE-F**	TTACCCACAGCGAGGGGATA	123
**OST48_DANRE-R**	AGGTGTTGGTATGACGGCTG	
**TBB_STRPU-F**	GAAGATAGCGGCGACTGTCA	121
**TBB_STRPU-R**	AAGTCAGCAGTACCGTGCTC	
**ATPA_PONAB-F**	TCCAAGAGCATCAACGACCC	150
**ATPA_PONAB-R**	GCTCTGAACTTGGAAGCCGA	
**ZN709_HUMAN-F**	CTTCGTGCTGTAGGAGGGTC	134
**ZN709_HUMAN-R**	CAAGGTCTGCGGAAAGGGAT	
**TBA_LEPDS-F**	ACCTGTGATCAGTTGCTCGG	173
**TBA_LEPDS-R**	CCGAGAAAGCGGTTGGATCT	
**RS5_HUMAN-F**	CCACGGCTGGTGGTAAGAAA	182
**RS5_HUMAN-R**	CAATCCGACAGATGGCGACT	
**Housekeeping**		
**MTB2-F**	GTGAATCCTGTACTGGCCTA	
**MTB2-R**	TGGTGAGAGGATACAGGAAA	120
